# Experience with a context-specific modified WHO safe childbirth checklist at two tertiary care settings in Sri Lanka

**DOI:** 10.1186/s12884-018-2040-6

**Published:** 2018-10-20

**Authors:** Hemantha M. Senanayake, Malitha Patabendige, Rathigashini Ramachandran

**Affiliations:** 1 Department of Obstetrics and Gynaecology, Faculty of Medicine, University of Colombo and University Obstetrics Unit, De Soysa Hospital for Women, Colombo, Sri Lanka; 2 University Obstetrics Unit, Teaching Hospital, Mahamodara, Galle, Sri Lanka; 3 University Obstetrics Unit, De Soysa Hospital for Women, Colombo, Sri Lanka

**Keywords:** WHO, Safe childbirth checklist, Implementation, Sri Lanka

## Abstract

**Background:**

The aim of the study was to assess whether a more context-specific modified version of WHO Safe Childbirth Checklist (mSCC) would result in improved adoption rate.

**Methods:**

A prospective observational study was conducted in University Obstetrics Unit at De Soysa Hospital for Women (DSHW), Colombo and two Obstetric Units at Teaching Hospital, Mahamodara, Galle (THMG), Sri Lanka. Study was conducted over 8 weeks at DSHW and over 4 weeks at THMG after introduction of the mSCC in 2017. The WHO SCC was in use at DSHW from 2013 until its replacement by the mSCC. Checklists were kept attached at admission and collected on discharge. Level of acceptance was assessed using a self-administered questionnaire at the end. Outcome measures were adoption rate (percentage of deliveries where mSCC was used and could be found), adherence to practices (mean percentage of items checked), response rate (percentage of staff members responded to questionnaire) and level of acceptance (percentage of “strongly agree/agree” in Likert scale to five questions regarding acceptance of mSCC). Responses were also taken to the open-ended question on barriers to implementation.

**Results:**

In DSHW, out of 606 births during study period, there were 329 live births in which the mSCC was used and could be found giving an adoption rate of 54.3%. In THMG adoption rate was 153/814 (18.8%). In DSHW, response rate for the questionnaire was 40.5% and in THMG, 40.0%. Level of acceptance was good among those who responded to the questionnaire. Mean (95% CI) adherence to the Checklist practices was 52.7% (44.1–58.5) in DSHW and 32.2% (24.5–39.1) in THMG with a range of 1–100% in both settings. Majority mentioned the lack of staff, lack of enthusiasm, inadequate training and advice on use of mSCC and lack of supervision from Ministry/institutional level. Majority suggested the involvement of medical doctors, removal of the need to place the signature and separate accountability to each 27-items and the desirability of proper training sessions regarding the mSCC.

**Conclusion:**

Checklist-based interventions in maternity care cannot be expected to improve by merely making them context-specific. Other approaches should be explored to maximize its benefits.

**Electronic supplementary material:**

The online version of this article (10.1186/s12884-018-2040-6) contains supplementary material, which is available to authorized users.

## Background

Reducing childbirth-associated mortality is a top global priority. There are more than 130 million births in the world each year. These result in an estimated 287,000 maternal deaths [1], 1 million intrapartum stillbirths [2] and 3 million newborn deaths [3]. The global the Maternal Mortality Rate (MMR) has fallen by 44% over the past 25 years, to an estimated 216 per 100,000 live births in 2015. Approximately 99% (302000) of these deaths occur in developing countries and m ost of these would have been prevented with timely, effective interventions [2, 3].

Skilled attendance at birth is a cornerstone of safe motherhood [4]. This has led to concerted efforts to motivate women in regions with a high MMR to deliver in healthcare facilities [5]. These efforts have led to a rise in institutional births [6], but the anticipated fall in morbidity and mortality has failed to materialize [7].

Poor quality care during institutional births in low and middle-income countries has been recognized to be a major contributory factor for childbirth-related harms [8]. Although skilled attendance may be available in healthcare facilities, they may fail to adhere to accepted protocols due to the failure to remember critical steps and the sequence in which to correctly execute them. A simple checklist that focuses on major causes of maternal mortality and morbidity could overcome these failures [9].

Identifying this need, the World Health Organization (WHO) designed the Safe Childbirth Checklist (SCC) [10, 11]. Its newest version is a list of 27 evidence-based critical practices, organized into four sections, each beginning with a ‘pause point’ [11]. The SCC has been developed particularly for low and middle-income settings, although it has been used high-income settings as well [12]. Items on the WHO SCC addresses major global causes of maternal death, such as haemorrhage, infection, obstructed labour and hypertensive disorders and fetal complications such as prematurity, intrapartum stillbirths and neonatal deaths [12].

Despite being an under-resourced country, Sri Lanka has achieved much in human development. This is reflected in the reduction in the MMR from 72.4 per 10,000 live births in 1995 to 32.0 per 100,000 live births in 2015 [13]. This is the lowest maternal mortality ratio in South Asia [14]. However, the MMR during the past 8–9 years has been stagnant despite an institutional delivery rate of over 99% [14]. Issues of quality of care may underlie this situation.

Our previous study conducted at the University Obstetric Unit of De Soysa Hospital for Women (DSHW), Colombo, Sri Lanka demonstrated poor adoption of SCC during births (45.8% of all deliveries) [15]. However, attitudes among healthcare workers towards its acceptance were positive (over 90%). This study also showed that provision of essential childbirth-related care practices at each birth was on average 21 out of 29 (95% CI 20.2; 21.3) [15]. We concluded that a follow-up study may be of value to further study the gap between poor adoption and high acceptance [15]. We felt that the gap between adoption and acceptance may have been due to the sCC not being context-specific for Sri Lanka. For example, Sri Lanka is a low-incidence country for human immunodeficiency virus (HIV) infection and the boxes related to the illness were hardly ever checked during the previous study [15]. Revision of the SCC with the help of national experts to ensure a context-specific adaptation in accordance with national standards, guidelines and culture is recommended by WHO [16]. In the hope of addressing weaknesses that may have contributed to the low adoption rate, we obtained inputs from stakeholders and prepared a modified version of WHO SCC.

This study was conducted to assess if a more context-specific modified SCC (mSCC) would result in an improved adoption rate.

## Methods

A hospital-based, prospective observational study was carried out in Sri Lanka in the University Obstetrics Unit of DSHW, Colombo and two Obstetric Units in the Teaching Hospital, Mahamodara, Galle (THMG), two busy tertiary care maternity hospitals in Sri Lanka. The investigators had experience in implementing the WHO SCC in DSHW from 2013, where the study was carried out over 8 weeks. At THMG, it was carried out over 4 weeks after introduction of the mSCC in 2017. For THMG it was the first exposure to a Safe Childbirth checklist. In the DSHW the WHO SCC (without modifications) was in use since 2013. The following common implementation model was applied to both settings.

Before the introduction of the intervention, the investigators gave the necessary basic education to healthcare workers. This consisted of imparting knowledge about the components of mSSC, its relevance to patient safety and quality improvement and how and when to use it. A copy of the WHO Safe Childbirth Checklist Implementation Guide [12] was used as a bedside tool and small groups [3–5] of staff members were trained using it. The staff was advised to mark the mSCC items in parallel to the practice of each item, optimizing the value of a checklist in clinical practice. Authors further reinforced their knowledge and attitudes by direct coaching and observations with twice-weekly sessions using at least 03–05 mothers in the first month and then with weekly sessions. All these steps were undertaken by authors RR at DSHW and MP at THMG.

In the mSCC, we removed sections that were least used in the previous study and added ones that were deemed important in the local context. We added the use of antenatal corticosteroids and encouraging the presence of a labour companion to the checklist. We also changed the first two ‘pause points’ – the first being advanced to the point of admission of the woman to the antenatal ward and the second to the point of admission to the labour ward. The mSCC was kept attached to clinical notes of every mother from admission to the ward to the point of discharge when they were collected into a separate file.

Outcome measures were adoption rate, adherence to practices, response rate and the level of acceptance. The adoption rate was taken as the percentage of deliveries where the mSCC was used during the study period. Adherence to checklist practices was calculated as a mean percentage of each item checked in mSCC out of the total in each setting. Successful adherence meant that the item was checked on the checklist.

The level of acceptance was assessed using a self-administered, pre-tested anonymous questionnaire consisting of two sections administered to all staff involved at the end of the study period (8 weeks in DSHW and 4 weeks in THMG). The response rate was the percentage of healthcare providers who responded to this questionnaire. The questionnaire included a five-point Likert scale for five stems focusing on the level of acceptance of SCC use and one open-ended question on the barriers to its use. The answers ‘strongly agree’ and ‘agree’ from the Likert scale were taken as satisfactory levels of acceptance and presented as percentages. Data analysis was carried out using standard statistical methods. Measures of dispersion and 95% confidence intervals were calculated. Ethical aspects of this study were reviewed by the Ethical Review Committee of the (EC-16-108), Faculty of Medicine, University of Colombo, Sri Lanka, which granted approval. Informed written consent was obtained from each participating staff member before giving the questionnaire.

**Fig. 1:**
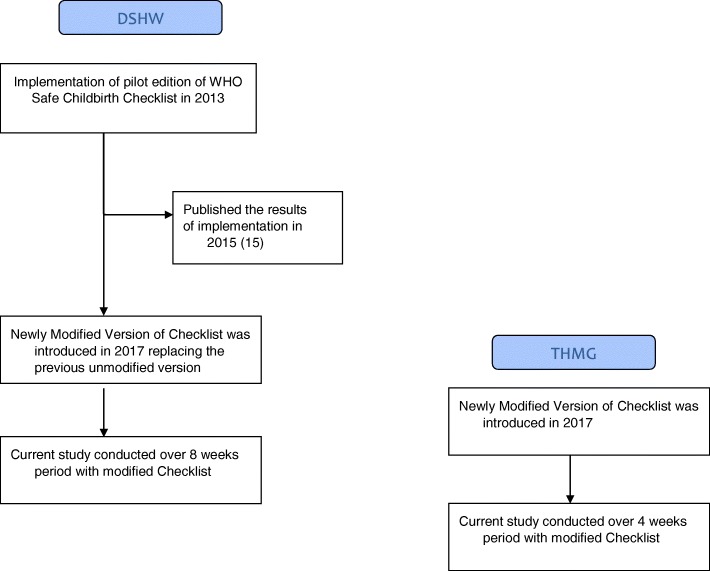
Sequential events of the WHO Safe Childbirth Checklist Implementation Programme in Sri Lanka

The basic flow chart of the process involved in the study is summarized in Fig. 1.

## Results

Basic demographic details of participating health workers are summarized in Table 1. Main outcome measures are summarized in Table 2. Table 3 shows adherence of the health care providers to each of the 27-items in the mSCC and in Table 3, a completed item indicates the percentage of women who were checked on the checklist for requiring the particular task. Table 4 shows responses to the questionnaire regarding attitudes of the care providers.

**Table 1:** Basic demographic details of participated health workers in the study with the newly modified version of WHO Safe Childbirth Checklist at both settings

Details of participating health workers	Mean age (SD)		Mean years of work experience (SD)		DSHW *n* = 30	THMG *n* = 36
	DSHW	THMG	DSHW	THMG	Frequency (%)	Frequency (%)
Nurses	40 (8.0)	37.6 (8.5)	17 (5.2)	11.8 (8.4)	18 (60.0)	20 (55.6)
Midwives	36 (1.8)	43.0 (2.1)	12 (2.0)	16.5 (0.5)	12 (40.0)	08 (22.2)
Doctors	0	29 (0.2)	0	1 (0.1)	0	08 (22.2)

*SD* Standard deviation, *DSHW* De Soysa Hospital for Women, *THMG* Teaching Hospital Mahamodara Galle

**Table 2:** Main outcome measures of the study with the newly modified version of WHO Safe Childbirth Checklist in both settings

Hospital	Average annual births	Number of births during study period	Number of deliveries in which mSCC had been used	Adoption rate, %	Total number of staff	Response rate, %	Mean (95% CI) adherence to Checklist practices, %
DSHW	4000	606	329	54.3	74	40.5	52.7 (44.1–58.5)
THMG	9000	814	153	18.8	90	40.0	32.2 (24.5–39.1)

Adoption rate: Percentage of deliveries where modified Checklist was used, Response rate: Percentage of staff members responded to the questionnaire, Adherence: Mean percentage of checklist items checked out of total, *DSHW* De Soysa Hospital for Women, *THMG* Teaching Hospital Mahamodara Galle, *mSCC* Modified Safe Childbirth Checklist, *CI* Confidence Interval

**Table 3:** Adherence (mean percentage of checklist items checked out of total) to each 27-items in modified WHO Safe Childbirth Checklist in both settings

Checklist item in modified WHO SCC	Adherence to each item (%)	
	DSHW (*n* = 329)	THMG (*n* = 153)
On admission to Antenatal ward		
1. Does mother need referral?	127 (38.6)	99 (64.7)
2. Does mother need to start antibiotics?	121 (36.8)	105 (68.6)
3. Does mother need to start antihypertensives/magnesium sulfate?	120 (36.5)	105 (68.6)
4. Does mother need corticosteroids?	123 (37.4)	105 (68.6)
5. Was mother informed about a birth companion?	118 (35.8)	108 (70.6)
6. Was she informed about PPIUD?	118 (35.8)	105 (68.6)
On admission to labour room		
7. Partogram started?	240 (72.9)	48 (31.4)
8. Does mother need antibiotics?	247 (75.1)	42(27.5)
Just before pushing (or before caesarean section)		
9. Does mother need to start magnesium sulphate/antihypertensives?	236 (71.7)	39 (25.5)
10. Confirm essential supplies are at bedside		
: For mother	237 (72.0)	36 (23.5)
: For baby	236 (71.8)	36 (23.5)
11. Informing Paediatric House Officer	240 (73.0)	33 (21.6)
Soon after birth (within 1 h)		
12. Maintenance of MEOWS chart	237 (72.0)	30 (19.6)
13. Does mother need antibiotics?	238 (72.3)	36 (23.5)
14. Does mother need to start magnesium sulphate/antihypertensives?	233 (70.8)	36 (23.5)
15. Continuous monitoring of neonate done	232 (70.5)	36 (23.5)
16. Does baby need referral?	233 (70.8)	36 (23.5)
17. Does baby need antibiotics?	225 (68.4)	33 (21.6)
18. Started breastfeeding and skin-to-skin contact	232 (70.5)	36 (23.5)
Before discharge		
19. Confirm stay at ward for 24 h after delivery	101 (30.7)	21 (13.7)
20. Does mother need antibiotics?	125 (38.0)	33 (21.6)
21. Is mother’s blood pressure normal?	124 (37.7)	33 (21.6)
22. Is mother bleeding abnormally?	123 (37.4)	33 (21.6)
23. Vaginal exam performed and checked for missing swabs/clots/ infected lochia	120 (36.5)	33 (21.6)
24. Does the baby need antibiotics?	121 (36.8)	33 (21.6)
25. Is baby feeding well?	120 (36.5)	33 (21.6)
26. Discuss and offer family planning options to mother	114 (34.7)	24 (15.7)
27. Arrange follow-up and confirm mother/companion will seek help if danger signs appear after discharge	52 (15.8)	15 (9.8)

A completed item indicates the percentage of women who were checked on the Checklist for requiring the particular task

*WHO* World Health Organization, *SCC* Safe Childbirth Checklist, *DSHW* De Soysa Hospital for Women, *THMG* Teaching Hospital Mahamodara Galle, *PPIUD* Postplacental Intrauterine Device, *MEOWS* Modified Early Obstetric Warning Score

**Table 4:** Attitudes for acceptance of using modified WHO Safe Childbirth Checklist in routine practice among participated health workers in both hospitals

Acceptance of using SCC in routine practice	Frequency (%)	
	DSHW (*n* = 30)	THMG (*n* = 36)
1. Modified WHO SCC should be made mandatory in Sri Lanka. (Strongly agree or Agree)	30 (100.0)	36 (100)
2. Modified WHO SCC improves inter-personal communication and team work among staff. (Strongly agree or Agree)	28 (93.3)	32 (88.9)
3. Modified WHO SCC helps to improve the quality of perinatal care. (Strongly agree or Agree)	30 (100.0)	32 (88.9)
4. If you or your family member is undergoing childbirth, should this modified WHO SCC be used. (Strongly agree or Agree)	29 (96.7)	36 (100)
5. Using the Modified WHO SCC is practical in Sri Lanka. (Strongly agree or Agree)	30 (100.0)	32 (88.9)

*WHO* World Health Organization, *SCC* Safe Childbirth Checklist, *DSHW* De Soysa Hospital for Women, *THMG* Teaching Hospital Mahamodara Galle

The adoption rates remained low across both sites (54.3% in DSHW & 18.8% in THMG). As shown in Table 1, in DSHW overall adherence in the labour room was satisfactory (more than 70%) compared to antenatal and postnatal wards (below 50%). We found similar results for the response rates in both settings (40.5% in DSHW and 40.0% in THMG). Checklist items that had to be filled in antenatal ward [1–6] and postnatal ward [19–27] were least checked (less than 50%) in DSHW, but the adherence rate for the criteria to be filled in labour room was above 70%. Checklist items which had to be filled in the labour room [7–18] and postnatal ward [19–27] were checked least in THMG (Table 2), reaching less than 25% overall. However, a vast majority of staff members who responded to the questionnaire accepted that using a checklist was a practical option (100.0% in DSHW and 88.9% in THMG). All who responded stated that they would like to see the SCC being made mandatory and almost all wanted it used in case the woman delivering was herself or a family member. As indicated in Table 4, attitudes towards acceptance of using the mSCC among health workers were satisfactory. Further, among the responders, 93% in DSHW and 88.9% in THMG agreed that mSCC stimulates inter-personal communication and teamwork among nurses, midwives, and doctors.

As responses to the open-ended question on barriers for implementation in our setting, the majority mentioned the lack of staff, lack of enthusiasm, lack of proper accountability to checklist items, inadequate training and advice on its use and lack of supervision from Ministry/institutional level. The majority suggested the increased involvement of medical doctors, removal of the need to place the signature and separate accountability to every 27 items and the desirability of proper training sessions regarding the SCC. Moreover, at THMG doctors emphasized that there is poor enthusiasm towards the use of SCC rather than lack of staff or high workload.

## Discussion

The main aim of this study was to evaluate if a more context-specific SCC would improve its utilization in healthcare facilities. However, the difference between the adoption rates, (54.3% in DSHW Versus 18.8% in THMG) could be attributed to the short duration of exposure to the ‘Safe Childbirth Checklist Concept’ in THMG (4 weeks) compared to the DSHW which had been exposed to it over a longer period. In the previous study in 2014, the adoption rate was 40.5% in DSHW [15]. Though this is a statistically significant increase in adoption rate in DSHW (*p* < 0.01), in real terms of coverage, it is still unsatisfactory. These results show that checklist-based interventions in maternity care cannot be expected to improve by merely making them context-specific. Other approaches need to be explored.

Although we have not studied it in detail, our findings demonstrate a probable dichotomy in the staff of both facilities. Those who responded to the questionnaire were enthusiasts who adopted the mSCC for the women who were under their care and those who did not respond were those who did not use the mSCC. This gap highlights the importance of the proper introduction, training, and coaching when introducing this tool in any new setting in accordance with WHO Safe Childbirth Checklist Implementation Guide [12].

Both of these research settings were busy tertiary level units with a varying complexity of cases, referrals and, transfers. These factors could make the staff more inclined to prioritize other activities rather than filling a checklist. It was also possible that staff in tertiary care units may have a high level of self-confidence and may have felt that a checklist was not required for them to discharge their duties to a high level. The majority who responded to the questionnaire accepted that the mSCC improved inter-personal communication and teamwork among different staff categories (Table 4).

In DSHW checking the item on encouraging a birth companion to be present was checked 35.9% of the time, which was almost half of what was seen in the previous study in 2014 (73.9%). In a unit that encourages birth companions, not discussing this aspect more often is not acceptable. However, only a few mothers were making use of the facility to have a birth companion despite it being encouraged. In THMG, this facility is not established even though women may have been made aware of it at antenatal clinics. Therefore staff has checked this item in more than 70% of cases and told them that this is not allowed at THMG. Checking of early commencement of breastfeeding was almost identical (70.52%) to the previous study (68.7%).

Checklist items that had to be filled in the labour room were checked less often in THMG (less than 25%) than that of DSHW (more than 70%). The low adherence to labour ward practices in THMG is a matter for concern since it is here that the mSCC was expected to have its greatest impact.

Most of the tertiary level birth facilities in Sri Lanka are functioning based on practical knowledge of care providers. This point was mentioned in response to the open-ended question in the questionnaire. There is a high risk of errors with this method as in ‘Reason’s Swiss Cheese Model’ [17] due to increased workload in under-resourced countries. Thus, a checklist could be important to minimize the errors and to improve the quality of care.

There could be many reasons for the low adoption rates seen in this study despite the checklist being made context-specific. Firstly, the care providers saw this intervention as a research undertaking, rather than a regulatory requirement. In a busy setting, there was no compulsion for the staff to use the mSCC. Secondly, some of the activities of the SCC were a duplication of what had to be filled in the official bed head ticket, which is the legally binding document. That would have taken priority over the mSCC. Thirdly, we may have given the care providers too many boxes to check. For example, the boxes regarding severe preeclampsia appear in three places. In the Sri Lankan setting, in hindsight, we felt it could have been confined to the first ‘pause-point’.

Given the findings of this study, we feel that the best way forward to implement the SCC would be a two-pronged approach. First, the boxes to be checked should be minimized, depending on the local context. We believe it should also take into account the potential impact - for example, the third ‘Pause-point’ could be advanced to 30 min after childbirth, where the height of the uterine fundus, the presence or absence of bleeding, the pulse rate and blood pressure are assessed by two workers in a true checklist fashion. In this way, the ‘golden hour’ is not lost and the most preventable and commonest single cause of maternal death could be treated without delay. Second, it needs to be incorporated into the official documentation of the healthcare system, taking into account the workflow of managing childbirth. That way, duplications could be circumvented and the maximum impact of the SCC could be ensured.

Importantly, there are certain limitations in this study that need to be highlighted when interpreting results. Our main goal was to test the modified version of the SCC and not its implementation program. This might have had an impact on the results. This is an observational study without a control group and data was collected from a self-administered questionnaire. The data in this study may be more specific to Sri Lanka, where the standard of care is of a better quality compared to most developing countries [14]. Moreover, looking at checklists that were filled out could overestimate or underestimate its use. It is possible that the checklists were simply filled out after delivery or at discharge and not in real time. It is also possible that some used the mSCC, without filling it out. Impact of these has to be considered as a major limitation when interpreting the results. Another significant limitation is the discrepancy in time periods of the study period (8 weeks in DSHW and 4 weeks in THMG). Even though authors reinforced their knowledge and attitudes using the Implementation Guide [12] from time to time, this step does not involve a direct unbiased observations. This is an important limitation. When compared to the previous studies from sites in the world which have been conducted with well-planned coaching-based interventions [10, 18, 19], this study has been conducted with a relatively light-touch intervention. Therefore it has to be acknowledged that the implementation model in this study was a significant limitation. Our availability of resources, level of support from the Ministry level and financial constraints might have affected these.

## Conclusions

Our findings show that adaptation of the SCC to the local context by itself would not improve its use by staff. Other approaches must be explored to maximize its benefits and adoptions. These measures could include incorporating the SCC into the official documentation of the facility to minimize duplication of work and make its use a regulatory requirement. Attention must be given to keeping the items to be checked to a minimum. Due attention must also be given to creating awareness among staff regarding the value of a SCC. The attitudes of healthcare workers who responded to our questionnaires were largely negative, though in a minority this was very positive. This could be built on by conducting awareness campaigns.

## Additional file

Additional file 1: WHO Modified Safe Child Checklist (mSCC). (PDF 502 kb)

## Abbreviations

DSHW: De Soysa Hospital for Women
HIV: Human Immuno-deficiency Virus
MMR: Maternal Mortality Ratio
mSCC: Modified safe childbirth checklist
SCC: Safe Childbirth Checklist
THMG: Teaching Hospital Mahamodara, Galle
WHO: World Health Organisation
