# Supplementary material for: Experience with a context-specific modified WHO safe childbirth checklist at two tertiary care settings in Sri Lanka
Source: BMC Pregnancy Childbirth. 2018 Oct 20;18:411. doi: 10.1186/s12884-018-2040-6 (PMC6196018; doi:10.1186/s12884-018-2040-6)
Supplement: Supplementary file 1 — WHO Modified Safe Child Checklist (mSCC). (PDF 502 kb) [file 12884_2018_2040_MOESM1_ESM.pdf]

Modified WHO Safe Childbirth Checklist

PILOT STUDY – DE SOYSA MATERNITY HOSPITAL

Serial No……………….. Ward…………… BHT No……………….

Name…………………………………………… Age …………………… POG ……………. Parity …………………

Antenatal Care- Modified WHO Safe Childbirth Checklist

On Admission to Ward

1. Does mother need referral?	☐ Yes, organized<br>☐ No	If yes mention here:-
2. Does mother need to start:<br>• Antibiotics?	☐ Yes, given<br>☐ No	Ask for allergies before administration of any medication.<br>Give antibiotics to mother if any:<br>• Mother's temperature >38^{0}C<br>• History of foul-smelling vaginal discharge<br>• Rupture of membranes >18hrs
• Magnesium sulphate and/or antihypertensive treatment?	☐ Yes, magnesium sulphate given	Give magnesium sulphate to mother if any of:<br>• Clonus<br>• Severe headache<br>• Visual disturbances and dizziness
	☐ Yes, antihypertensive given	Give antihypertensive medication to mother if:<br>• Systolic BP >160mmHg<br>• Diastolic BP >110 mmHg<br>• Mean arterial pressure >/= 125mmHg
	☐ No	Aim to maintain BP at around 130-140/90-100 mmHg
3. Does she need antenatal corticosteroids?	☐ Yes, given<br>☐ No	Start antenatal corticosteroids Preterm birth, gestation age between 24-34 weeks<br>• Mothers with PPROM<br>• Mothers with hypertension<br>• Mothers with diabetes<br>• Mothers with foetal growth restriction
4. Has the mother informed about a birth companion?	☐ Yes. Informed<br>☐ No	If yes, is labour companion informed?<br>☐ Yes ☐ No
5. Has she informed about PPIUD?	☐ Yes<br>☐ No	If yes, is consent form signed:-<br>☐ Yes ☐ No

Completed by…………………………… Date ………………………

Modified WHO Safe Childbirth Checklist

PILOT STUDY – DE SOYSA MATERNITY HOSPITAL

Intrapartum Care

On Admission to Labour room

6. Partogram started?	☐ Yes<br>☐ No, will start when >4cm	Start plotting when cervix >/=4cm, and cervix should dilate >/= 1cm/hr
7. Does mother need antibiotics?	☐ Yes<br>☐ No	If yes, mention the indication:-

Just before Pushing (or Caesarean section)

8. Does mother need:<br>• Antibiotics?	☐ Yes, given<br>☐ No	Ask for allergies before administration of any medication. Give antibiotics to mother if any:<br>• Mother's temperature >38^{0}C<br>• History of foul-smelling vaginal discharge<br>• Rupture of membranes >18hrs<br>• Caesarean section
• Magnesium sulphate and antihypertensive treatment?	☐ Yes, magnesium sulphate given<br>☐ Yes, antihypertensive given<br>☐ No	} Please refer page 1
9. Confirm essential supplies are at bedside and prepare for delivery	☐ For mother:<br>• Gloves<br>• Alcohol based hand rub or soap and clean water<br>• Delivery set<br>• Oxytocin 5 Units in Syringe<br><br>☐ For baby:<br>• Clean towel<br>• Sterile blade/scissors to cut cord<br>• Switch on warmer<br>• Bag-and-mask checked for function	Prepare to care for mother immediately after birth:<br>1. Confirm single foetus only<br>2. Give oxytocin immediately after the birth of the baby<br>3. Controlled cord traction to deliver placenta<br>4. Uterus massage after placenta is delivered<br><br>Prepare to care for baby immediately after birth:<br>1. Dry baby and keep warm<br>2. If not breathing: stimulate and clear airway<br>3. If still not breathing<br>− Cut cord<br>− Ventilate with bag-and-mask<br>− Shout for help
10. PHO informed if necessary	☐ Yes, informed<br>☐ No	PHO should present before delivery in following circumstances<br>1. Instrumental delivery<br>2. Meconium stained liquor<br>3. Twin delivery<br>4. Caesarean<br>5. Diagnosed with congenital anomalies (Diaphragmatic hernia, Heart diseases)<br>6. Delivery at POA <34 Weeks

Completed by.................................   Date ....

Modified WHO Safe Childbirth Checklist

PILOT STUDY – DE SOYSA MATERNITY HOSPITAL

Intrapartum Care

Soon after birth (within 1 hour)

11. Continuous monitoring of maternal status:<br>• MEOWS chart commenced	☐ Yes<br>☐ No	Inform House Officer if mother has any of:<br>1. Continuing fresh bleeding<br>2. Elevation of the level of the fundus<br>3. Increase of pulse rate above 100 or by 30 beats per minutes<br>4. Drop in systolic blood pressure below 100 or by 30mmHg<br>5. Blood loss of >1000ml<br>6. Deterioration of the patient out of proportion to the overt blood loss
12. Does mother need:<br>• Antibiotics?	☐ Yes, given<br>☐ No	Ask for allergies before administration of any medication. Give antibiotics to mother if placenta manually removed of if Mother's temperature >38^{0}C and if any:<br>• Chills<br>• History of foul-smelling vaginal discharge<br><br>If the mother has a third or fourth degree of perineal tear give antibiotics to prevent infection
• Magnesium sulphate and antihypertensive treatment?	☐ Yes, magnesium sulphate given<br>☐ Yes, antihypertensive given<br>☐ No	Please refer page 1
13. Continuous monitoring of newborn status:<br>• Neonatal Assessment	☐ Yes<br>☐ No	Inform House Officer if new born has any of:<br>1. Respiratory rate >60/min with retractions<br>2. Chest in-drawing, grunting, or Apnoea<br>3. Difficulty in arousal, coma<br>4. Baby temperature <35.5^{o}C (and not rising after warming) or baby's temperature >37.50^{o}C<br>5. Central cyanosis<br>6. Shock (cold periphery, CRT>3sec, rapid thready pulse)
14. Does baby need:<br>• Referral	☐ Yes, organized<br>☐ No	If yes mention the indication:-
• Antibiotics	☐ Yes, given<br>☐ No	If yes mention the indication:-
15. Started breastfeeding and skin-to-skin contact (If baby and mother well)	☐ Yes, arranged<br>☐ No	

Completed by…………………………… Date ………………………

Modified WHO Safe Childbirth Checklist

PILOT STUDY – DE SOYSA MATERNITY HOSPITAL

Postnatal Care

Before Discharge

16. Confirm stay in ward for 24 hours after delivery ☐Yes ☐No		
17. Does mother need antibiotics?	☐ Yes, given and delayed discharge<br>☐ No	Ask for allergies before administration of any medication. Give antibiotics to mother if any:<br>• Mother's temperature >38^{0}C<br>• History of foul-smelling vaginal discharge
18. Is mother's blood pressure normal?	☐ Yes<br>☐ No, treated and delayed discharge	Please refer page 1
19. Is mother bleeding abnormally?	☐ Yes, treated and delayed discharge<br>☐ No	If pulse >110 beats per minute and blood pressure <90mmHg<br>• Start IV and keep mother warm<br>• Treat cause (hypovolaemic shock)
20. vaginal examination done and checked for missed swabs?	☐ Yes<br>☐ No	
21. Does the baby need antibiotics?	☐ Yes, given antibiotics, special care given and delayed discharge<br>☐ No	Inform House Officer if new born has any of:<br>1. Respiratory rate >60/min with retractions<br>2. Chest in-drawing, grunting, or Apnoea<br>3. Heart rate >/=160bpm, low pulse volume, hypotension, shock<br>4. Difficulty in arousal, coma<br>5. Baby temperature <35.5^{o}C (and not rising after warming) or baby's temperature >37.50^{o}C<br>6. Stopped breastfeeding well<br>7. Umbilical redness extending to skin or draining pus
22. Is baby feeding well?	☐ Yes<br>☐ No, established good breastfeeding practices and delayed discharge	Cannot discharge if either both of the following not satisfactory<br>1. Weight loss >5% on day 1<br>2. Positioning and technique of breast feeding (checked and documented as "satisfactory/not" by qualified trained doctor/ lactation nurse or midwife)
23.Discuss and offer family planning options to mother	☐ Yes, offered and arranged<br>☐ No	
24. Arrange follow-up and confirm mother/ companion will seek help if danger signs appear after discharge ☐Yes ☐No		

Danger signs

Mother has any
- ☐ Bleeding
- ☐ Severe abdominal pain or epigastric pain
- ☐ Severe headache or visual disturbances
- ☐ Breathing difficulty
- ☐ Fever or chills
- ☐ Difficulty in emptying bladder

Baby has any
- ☐ Fast/ difficult breathing
- ☐ Fever
- ☐ Unusual cold
- ☐ Stop feeding well
- ☐ Less activity than normal
- ☐ Whole body become yellow

Completed by…………………………… Date ……………………..
